# Vestibular System and Self-Motion

**DOI:** 10.3389/fncel.2018.00456

**Published:** 2018-11-22

**Authors:** Zhixian Cheng, Yong Gu

**Affiliations:** ^1^Department of Neuroscience, Yale School of Medicine, New Haven, CT, United States; ^2^Key Laboratory of Primate Neurobiology, CAS Center for Excellence in Brain Science and Intelligence Technology, Institute of Neuroscience, Chinese Academy of Sciences, Shanghai, China

**Keywords:** vestibular, self-motion perception, heading, path trajectory, distance perception

## Abstract

Detection of the state of self-motion, such as the instantaneous heading direction, the traveled trajectory and traveled distance or time, is critical for efficient spatial navigation. Numerous psychophysical studies have indicated that the vestibular system, originating from the otolith and semicircular canals in our inner ears, provides robust signals for different aspects of self-motion perception. In addition, vestibular signals interact with other sensory signals such as visual optic flow to facilitate natural navigation. These behavioral results are consistent with recent findings in neurophysiological studies. In particular, vestibular activity in response to the translation or rotation of the head/body in darkness is revealed in a growing number of cortical regions, many of which are also sensitive to visual motion stimuli. The temporal dynamics of the vestibular activity in the central nervous system can vary widely, ranging from acceleration-dominant to velocity-dominant. Different temporal dynamic signals may be decoded by higher level areas for different functions. For example, the acceleration signals during the translation of body in the horizontal plane may be used by the brain to estimate the heading directions. Although translation and rotation signals arise from independent peripheral organs, that is, otolith and canals, respectively, they frequently converge onto single neurons in the central nervous system including both the brainstem and the cerebral cortex. The convergent neurons typically exhibit stronger responses during a combined curved motion trajectory which may serve as the neural correlate for complex path perception. During spatial navigation, traveled distance or time may be encoded by different population of neurons in multiple regions including hippocampal-entorhinal system, posterior parietal cortex, or frontal cortex.

## Introduction

Accurate and precise detection of displacement of our head and body in space is critical for important functions including balance, posture control, gait, spatial orientation and self-motion perception. It can be accomplished through the vestibular pathway that starts from two small but elegant organs embedded in our inner ears: the otolith and semicircular canals, which detect linear and angular acceleration of our head, respectively (Goldberg and Fernandez, 1971; Fernández and Goldberg, 1976a,b). The encoded inertial motion signals in the peripheral system are propagated to the central nervous system for further processing. While the neural circuits mediating automatic process such as vestibulo-ocular reflex (VOR) for maintaining visual stability and body balance are well known (Takakusaki, 2017), less is clear about how vestibular signals are coded by the brain for perception of self-motion and spatial orientation (Lopez, 2016). Recent neurophysiological studies have discovered that robust vestibular signals are distributed broadly in sensory cortices, suggesting that the vestibular system may be involved in higher cognitive functions (Gu, 2018).

In this review article, we summarized recent progress on the involvement of the vestibular system in higher cognitive functions, particularly for self-motion. We will focus on three topics: (1) how vestibular signals may contribute to estimating of one’s heading direction through space; (2) how a more complex path trajectory may be coded by convergent translation and rotation signals arising independently from the otolith and horizontal canals; and (3) how traveled distance or time is possibly coded by the vestibular system. For each topic, we first reviewed results from psychophysical studies on humans and monkeys, and then pointed to the neurophysiological studies that may provide insights for the underlying neural mechanisms. We finally discussed remained issues that need to be addressed in future studies. Note that we have focused primarily on recent progress in the cortical system. Numerous studies conducted in subcortical areas including thalamus, hippocampus, and the limbic system including the entorhinal cortex, retrosplenial cortex can be referred elsewhere, and are only briefly mentioned in the current review article.

## Heading

Both human and monkey can judge heading directions accurately and precisely based on vestibular cues (Telford et al., 1995; Ohmi, 1996; Gu et al., 2007; Fetsch et al., 2009, 2011; Crane, 2012; Drugowitsch et al., 2014). The intact vestibular system is crucial for heading estimation. For example, bilateral labyrinthectomy led to dramatic increase in the psychophysical threshold in a vestibular heading discrimination task, in which the monkeys were instructed to report their perceived heading directions delivered through a motion platform under a two-alternative-forced-choice experimental paradigm (Gu et al., 2007). However, psychophysical threshold decreased gradually after labyrinthectomy, suggesting that the animals may learn to use other sensory inputs, for example, somatosensory or proprioceptive cues to compensate the deficiency in the vestibular system. This hypothesis is consistent with the phenomenon that the animals began to lean their hands against the wall of the cage when moving around after labyrinthectomy. Note that, the psychophysical threshold remained about 10 times worse than the baseline (i.e., before labyrinthectomy), demonstrating that the function of the vestibular system could not be fully compensated by other sensory systems (Gu et al., 2007).

Other sensory inputs, in particular, visual cues, do help the vestibular system for more accurate and precise heading estimate. Provided with congruent vestibular and visual optic flow cues, both humans (Butler et al., 2010, 2011, 2015; Crane, 2017; Ramkhalawansingh et al., 2018) and monkeys (Gu et al., 2008; Fetsch et al., 2009; Chen et al., 2013) can judge smaller heading directions compared to the condition when only one sensory input is available. Interestingly, the decrement in psychophysical threshold during cue combined condition is consistent with the prediction from the optimal cue integration theory (Ernst and Banks, 2002), indicating that our brain makes full use of the information when summing sensory evidence from different sensory modalities. The optimal performance is verified under conditions when a conflict heading angle between vestibular and visual cues is introduced (Fetsch et al., 2009; Butler et al., 2015), or when subjects performed a reaction-time version of the task in which they do not have to wait and accumulate sensory evidence for a long and fixed duration (Drugowitsch et al., 2014).

The neural substrate for heading perception has been extensively explored within the last three decades. Most of the studies have largely focused on areas within the cerebral cortex because neurons in many of these areas are modulated by complex optic flow that is typically experienced during natural navigation. For example, Duffy and colleagues have shown that neurons in the dorsal portion of the medial superior temporal sulcus (MSTd) are sensitive to global-field optic flow simulating real self-motion (Duffy and Wurtz, 1991, 1995), as well as to transient whole body movement in darkness (Duffy, 1998; Page and Duffy, 2003). Later on, Angelaki and DeAngelis further characterized heading selectivity of MSTd neurons in three-dimensional (3D) space using a six degree of freedom (6-DOF) motion platform (Gu et al., 2006; Takahashi et al., 2007; Morgan et al., 2008). They found that nearly all MSTd neurons are significantly modulated by optic flow and two thirds are significantly tuned to vestibular stimuli. Labyrinthectomy largely diminished the vestibular activity but not visual activity in MSTd, suggesting the responses measured during the physical motion condition in darkness really arise from the vestibular source (Gu et al., 2007; Takahashi et al., 2007). Interestingly, for neurons significantly modulated by both optic flow and inertial motion, about half prefers congruent heading direction, and these “congruent” neurons typically exhibit higher heading selectivity when both cues are provided in a congruent way, constituting an ideal substrate for more robust heading estimate during natural navigation. However, note that the other half neurons tend to carry conflict visual and vestibular heading information, producing weaker heading selectivity during cue combination. Thus, this population of neurons is unlikely to account for more robust heading estimate under congruent vestibular-visual inputs. The exact functional implications of these neurons remain a mystery at this stage.

**Figure 1:**
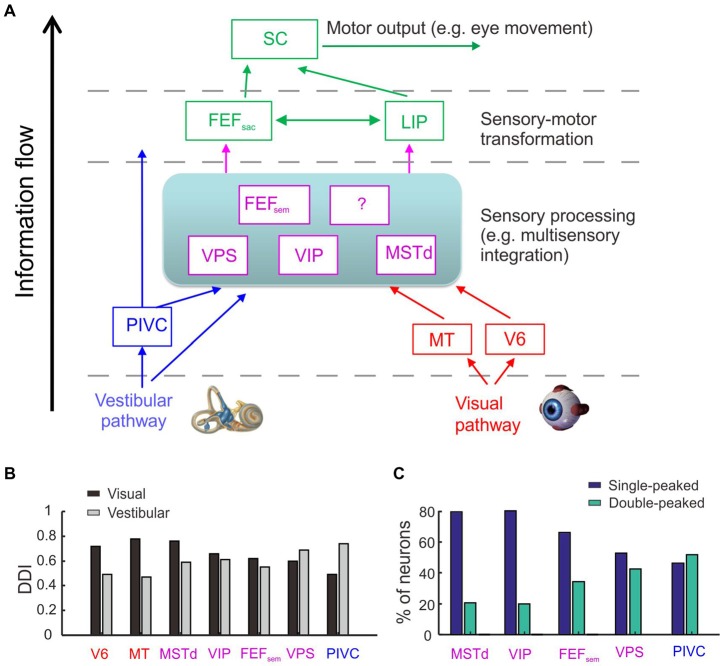
Cortical regions involved in heading perception and their spatial and temporal tuning properties. **(A)** Possible cortical network involved in heading perception revealed by recording neurons in macaques during translating the whole body using a motion platform system. Arrows represent possible information flow based on previous neurophysiological findings. PIVC, parieto-insular vestibular cortex; VPS, visual posterior sylvian area; VIP, ventral intraparietal area; MSTd, the dorsal portion of medial superior temporal area; FEF_sem_, smooth eye movement region of frontal eye field; FEF_sac_, saccade region of frontal eye field; V6, area V6; MT, middle temporal area; LIP, lateral intraparietal area. Blue: vestibular dominant area or pathway; Red: visual dominant area or pathway; Magenta: areas with converged visual and vestibular signals; Green: sensory-motor transformation areas involved in oculomotor decision tasks. **(B)** The spatial tuning strength quantified by a direction discrimination index (DDI). DDI value ranges from 0 to 1, with 0 indicating no selectivity and 1 indicating high selectivity (Takahashi et al., 2007). Gray: DDI values measured under the vestibular condition; Black: DDI values measured under the visual condition. Redrew using data from Fan et al. (2015) and Gu et al. (2016). **(C)** The temporal tuning property under the vestibular condition quantified by the proportion of single-peaked neuron (navy blue) and double-peaked neuron (spring green). Redrew using data from Chen et al. (2010, 2011a,b,c) and Gu et al. (2016). The temporal dynamics of the single-peaked neurons follow more closely with the velocity profile of the vestibular stimuli, whereas temporal dynamics of the double-peaked neurons match more with the acceleration profile.

Using the same paradigm, researchers have examined a number of areas in the cerebral sensory cortices and cerebellum. Many of these areas exhibit similar neuronal properties as those found in MSTd, including the ventral parietal area (VIP; Chen et al., 2011c), the smooth eye movement area of the frontal eye field (FEF_sem_; Gu et al., 2016), the visual posterior sylvian area (VPS; Chen et al., 2011b), and the cerebellar nodulus and uvula (Yakusheva et al., 2013). However, some areas exhibit different properties. For example, most neurons in the posterior insular vestibular cortex (PIVC) are only tuned to vestibular stimuli, but not to optic flow (Chen et al., 2010). By contrast, most neurons in the middle temporal area (MT; Chowdhury et al., 2009) and V6 (Fan et al., 2015) only respond to visual stimuli, but not to inertial motion. Taken together, we can sketch a map with each area serving as a node in the network for heading perception (Figure 1A; see review by Gu, 2018). Note in this map, sensory information is hypothesized to further transmit to decision-related areas such as the frontal and parietal lobes (e.g., FEF_sac_ and LIP) in which the evidence of sensory inputs is accumulated and transformed to form decision and generate motor output. How momentary vestibular evidence is accumulated during this process is still unverified. For example, is the vestibular acceleration accumulated for heading estimate (Drugowitsch et al., 2014)? Future physiological experiments need to be conducted to examine this hypothesis.

The exact information flow across the heading network is currently unknown. However, there are hints from some properties of the neurons recorded from these areas (Figures 1B,C). First, the strength of vestibular heading selectivity tends to increase from visual dominant areas (e.g., V6) to vestibular dominant areas (e.g., PIVC; Figure 1B). Second, the vestibular temporal dynamics are heterogeneous in the brain. In the peripheral otolith organs, vestibular signals predominantly encode the acceleration component of the inertial motion. Yet these signals are integrated more or less after propagating to the central nervous system, leading to temporal dynamics varied from acceleration to velocity dominant profiles (Laurens et al., 2017; see review by Gu, 2018). Across sensory cortices, the proportion of velocity dominant neurons tends to decrease gradually from area MSTd to PIVC, whereas the proportion of acceleration dominant neurons shows an opposite trend (Chen et al., 2011a). These results suggest that PIVC may lie most proximally to the vestibular periphery, followed by VPS and FEF_sem_, and then VIP and MSTd.

Although the vestibular and visual heading signals are broadly distributed within the brain network, it is unclear about which areas are really involved in heading estimate. Recently some studies were conducted to address this issue. In these studies, animals were required to actively report their experienced heading directions (Gu et al., 2007, 2008; Fetsch et al., 2009, 2011; Liu et al., 2010; Chen et al., 2013). At the same time, neural activities in certain areas were artificially manipulated to test their causal roles in heading perception. For example, researchers injected chemical drugs (such as muscimol) into the brain to suppress neuronal activity, and found that inactivation of PIVC greatly diminishes the animals’ heading performance based on vestibular cues, but not much in the optic flow condition (Chen et al., 2016). On contrary, inactivation of MSTd greatly diminishes the animals’ heading performance based on optic flow, but not much in the vestibular condition (Gu et al., 2012). These results suggest that the vestibular-dominant area PIVC plays a critical role in heading perception based on inertial motion, whereas the visual-dominant area MSTd is key to heading based on optic flow.

Different from PIVC and MSTd, inactivation of area VIP does not generate significant effects on the animals’ heading performance based on either the vestibular or visual cues (Chen et al., 2016). Such a result is surprising because VIP is similar to MSTd in many aspects (Britten, 2008; Maciokas and Britten, 2010). For example, both areas carry robust vestibular and visual heading signals (Chen et al., 2011c). In addition, neuronal activity in VIP also co-varies with the animal’s choice on a trial to trial basis, and this choice-correlation effect is even larger compared to that in MSTd (Chen et al., 2013; Zaidel et al., 2017; Yu and Gu, 2018). Hence, the exact functional implications of the motion directional signals in VIP remain unclear and require further investigation, probably by using other techniques or other behavioral paradigms. For example, by delivering weak electrical currents into the brain to selectively activate a cluster of neurons, researchers examine whether the animals’ perceptual judgments are biased in the direction that is predicted from the artificially stimulated neurons (e.g., Salzman et al., 1992). Such an effect implies that the examined area plays a sufficient role in the perceptual decision making task. Using this technique, researcher found that microstimulation in MSTd produces significant effects on the animals’ heading performance based on optic flow (Gu et al., 2012; Yu and Gu, 2018; Yu et al., 2018), but not for VIP (Yu and Gu, 2018). However, this effect becomes significant when smooth eye movements were simultaneously accompanied the presented heading stimuli (Zhang and Britten, 2011). In another study, electrical stimulation in VIP could even directly evoke complex facial movements (Cooke et al., 2003). Thus, compared to other sensory cortices (e.g., MSTd), VIP seems to carry more motor-related signals and may causally contribute to behavior only when more complex behavior is involved.

## Trajectory of Self-Motion

Our motion trajectory through the space can be complex, typically composed of both translation and rotation components rather than only one of them. For example, when animals run away from their predators, they may make turns while remain heading forward at the same time, resulting in a curved motion trajectory. Curved motion also frequently happens in human world, for example, vehicle driving, ski and running race in sports. How could complex motion trajectories be represented by the vestibular system?

Recent studies begin to address this issue by focusing on interactions of translation and rotation signals arising from otolith and semicircular canals respectively, particularly in the horizontal plane. For example, researcher have designed experiments in which human subjects were instructed to navigate along a curved motion trajectory through passive driving or active walking (Ivanenko et al., 1997; Israël et al., 2005; Nooij et al., 2016). The subjects were then required to reproduce the experienced path by drawing, walking or driving a vehicle. This is not a trivial task because to reproduce the exact profile of the experienced motion trajectory, the subjects need to discriminate the relative translation and rotation components over time during navigation (Li and Cheng, 2011). It showed that the blindfolded subjects were quite good at recovering the traveled path either under the straight or curved motion conditions, suggesting that similar to visual optic flow cues (Li and Cheng, 2011), vestibular signals could also be reliable enough for path perception. However, subjects could not effectively distinguish real curved self-motion from a straight motion trajectory accompanied by a yaw rotation of the head or whole body at the same time (i.e., illusorily perceived curved motion; Ivanenko et al., 1997; Israël et al., 2005). Thus, signals arising from horizontal canals seem to play a critical role in complex path perception. Indeed, a recent study examined the detection threshold for head translation and rotation respectively during combined, i.e., curvilinear motion (MacNeilage et al., 2010). It is found that the detection threshold for rotation was unaffected under the presence of translation, while the detection threshold for translation was significantly increased under the presence of rotation. In a different study, researcher found that yaw rotations could significantly bias the subjects’ perceived sway of the body, but a reversed effect did not happen (Crane, 2016). Finally, when asked to reproduce a triangle path, some patients with vestibular deficits could replicate the traveled distance, but not the traveled angle (Glasauer et al., 2002), indicating a causal role of the vestibular signals, particularly for the rotation signals in complex path perception.

**Figure 2:**
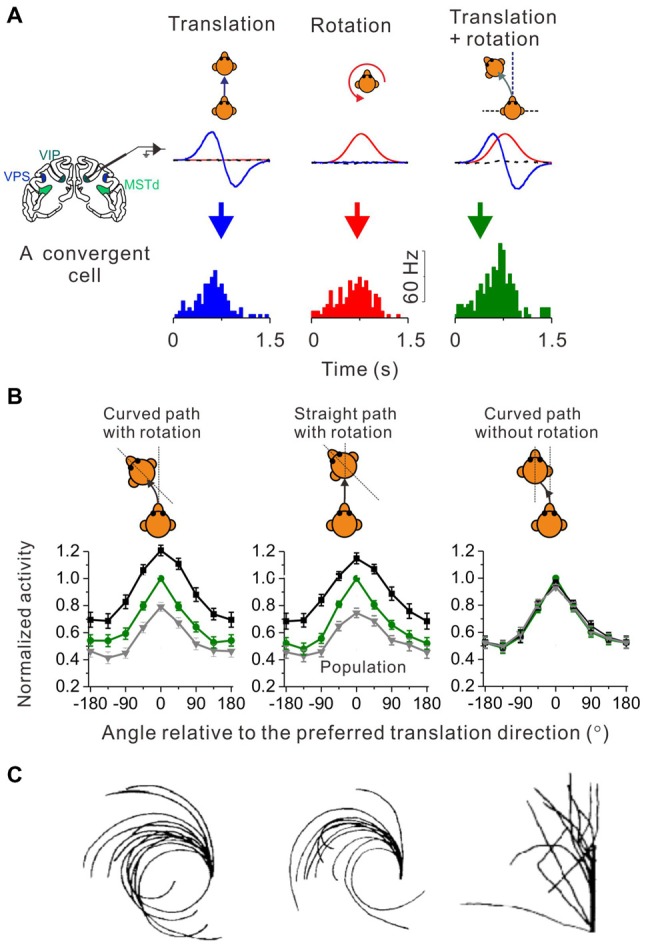
Identify cortical neurons responding to curvilinear self-motion. **(A)** Top panel: schematic illustration for three types of self-motion; middle panel: measured linear acceleration (Blue curve) and angular velocity (Red curve) for forward translation, CCW rotation and their corresponding curvilinear motion; Bottom panel: PSTH to forward translation, counter-clockwise (CCW) rotation and curvilinear motion with combined forward translation and CCW from an example convergent neuron in area VPS. **(B)** Firing rate pattern of convergent neurons from areas VPS, VIP and MSTd during curved-path-with-rotation, straight-path-with-rotation, and curved-path-without-rotation. Green curves: the translation only condition; black curves: curvilinear condition with preferred rotation; gray curves: curvilinear conditions with non-preferred rotation. Plots were made and modified with permission from Cheng and Gu (2016). **(C)** Trajectories drew by blinded-folded subjects after experiencing curved-path-with-rotation, straight-path-with-rotation and curved-path-without rotation delivered by a vehicle. Plots were made and modified with permission from Ivanenko et al. (1997).

How do neurons in the brain carry out computations that could underlie the curvilinear self-motion perception? To address this issue, researchers recorded single-unit activity from neurons in the central nervous system of macaques under translation only, yaw rotation only and convergent translation plus yaw rotation conditions. In the vestibular nucleus (VN) in brainstem, neurons integrate translation and rotation inputs in a sub-additive (Carriot et al., 2015) or near additive way (Newlands et al., 2018) when both signals co-exist in the curvilinear motion condition. Researchers also have examined several cortical areas including MSTd, VIP and VPS, and found that a group of convergent neurons receiving both translation and rotation inputs tended to integrate the two signals sub-additively (Figure 2), suggesting that this property may arise from the subcortical areas, e.g., the brainstem (Cheng and Gu, 2016). However, the weight assigned to the translation and rotation signals in cortices is not consistent with what has been reported in the brainstem, suggesting that additional integration may also happen when the vestibular signals are propagated to the cortex.

Curved motion trajectory in the horizontal plane would potentially produce centripetal force that may also mediate curvilinear self-motion perception. However, using a straight linear path with simultaneous head rotation paradigm (Ivanenko et al., 1997), researchers found that human subjects reported almost the same “curved” motion experience as in the curved path condition with head rotation (Figure 2C, middle panel vs. left panel). Because the magnitude of the centripetal force is quite different between these two experimental conditions, it is unlikely that the centripetal force would be a key to curved motion sensation. Indeed, in a third experimental conditions in which subjects experienced curved motion path but without head rotations, they did not report curved self-motion any more although the centrifugal force was now present as in the curved path condition with head rotation (Figure 2C, right panel vs. left panel). On the neural level, recently researcher recorded neurons in a number of cortical areas under a similar paradigm as in the above psychophysical study (Cheng and Gu, 2016). Interestingly, similar to the behavior, the firing patterns of the cortical neurons are analogous under the curved-path-with-rotation condition and straight-path-with-rotation condition, but are different from the condition when yaw rotation is absent (Figure 2B). Thus, neurons receiving inputs from both otolith and horizontal canals in the brain may mediate curvilinear self-motion perception. Note, that physiological properties including the proportion of different types of neurons, tuning strength and sensory summation rules are similar across the examined cortical areas, suggesting that the complex motion trajectory may be widely represented in the brain. However, future work is required to dissect the exact role of individual areas in self-motion.

## Travel Distance

From the mathematical point of view, double integration of the vestibular acceleration signals provides information about the distance we have traveled, which appears to be more challenging than estimating heading direction during spatial navigation. Researchers have investigated the role of vestibular signals in distance perception by requiring blindfolded human subjects to report their linear or angular displacement of the body through a number of methods including pointing (Ivanenko et al., 1997; Nooij et al., 2016), saccade (Berthoz et al., 1987; Israël and Berthoz, 1989), pressing button (Israël et al., 1993; Harris et al., 2000), walking (Mittelstaedt and Mittelstaedt, 2001; Campos et al., 2010, 2012, 2014), or controlling vehicles (Grasso et al., 1999; Tremblay et al., 2013). Normal human subjects could accurately recover their traveled distance, as well as the motion velocity profile regardless of reporting methods. By contrast, performance from vestibulopathy subjects were typically impaired in estimating time and distance when they were instructed to walk forward and make turns at a particular point under a blindfolded condition (Cohen, 2000), suggesting a causal role of vestibular signals in distance perception.

Similar to heading perception, information from other sensory modalities such as visual and proprioceptive cues, also contribute to the estimation of traveled distance (Jürgens et al., 2003; Jürgens and Becker, 2006). For example, a number of studies have illustrated that subjects can accurately estimate the traveled distance from optic flow (Bremmer and Lappe, 1999; Redlick et al., 2001; Frenz and Lappe, 2005; Dacke and Srinivasan, 2007). When different sensory inputs are provided at the same time, information from different sources is summed with a weight that is proportional to the reliability of each cue (Sun et al., 2004; Campos et al., 2010, 2012, 2014; ter Horst et al., 2015). However, some work proposed that the vestibular signals could dominate the visual signals (Harris et al., 2000), similar to a prior of the vestibular signals as observed in heading discrimination tasks (Fetsch et al., 2011; Butler et al., 2015).

Unlike the extensive studies exploring the contribution of vestibular signals in heading perception, little is known about the role of vestibular signals underlying distance perception. There is evidence suggesting that the temporoparietal junction, which carries prominent vestibular signals, may be involved in distance perception. For example, patients with lesions in the temporoparietal region tended to underestimate the traveled distance and stimulus duration, whereas the ability to detect onset of motion was unaffected (Kaski et al., 2016). When using repetitive transcranial magnetic stimulation (rTMS) to interfere the temporoparietal junction, subjects could replicate the motion velocity profile, but could not replicate the traveled distance (Seemungal et al., 2008a,b, 2009; Ventre-Dominey, 2014). This result suggests that temporoparietal junction plays an important role in distance perception, and moreover, distance perception (related to integration of velocity information over time) and heading perception (related to detection of motion direction over time) are two separate processes implemented in the brain.

How exactly vestibular signals in cortex contribute to the estimation of traveled distance or time remains unclear. Recently a study characterizing the spatial-temporal properties of vestibular responses in MSTd found that nearly half of the neurons exhibited a statistically significant position component, yet it was much weaker compared to the velocity as well as the acceleration component (Chen et al., 2011a). More works need to be conducted in the future to characterize how neurons in different cortical areas (see Figure 1) may encode the moving distance. For example, neurons in the sensory-motor transformation areas including the parietal and frontal lobes exhibit ramping activity over time (Kim and Shadlen, 1999; Gold and Shadlen, 2000; Shadlen and Newsome, 2001; Ding and Gold, 2012), which may serve as a neural correlate for distance coding. Indeed, it has been indicated that parietal neurons may encode the elapsed time (Jazayeri and Shadlen, 2015), thus, these neurons may also encode the traveled distance as the product of the time and moving speed. Such neurons have also been reported in subcortical areas such as the rodents’ hippocampus when the animals performed a spatial navigation task (Kraus et al., 2013).

In fact, via the anterior part of thalamus, vestibular peripheral inputs project to the limbic system which has been illustrated to be critical for self-motion based path integration (Cullen and Taube, 2017). For example, rotation signals arising from the semicircular canals are necessary for formation of head direction cells (Valerio and Taube, 2016). Translation signals from otolith may be critical for place cells, grid cells, and speed cells in the hippocampal-entorhinal system (Yoder and Kirby, 2014). It remains unclear how exactly the cortical self-motion system is connected with the subcortical and limbic systems, for example, through retrosplenial cortex (Vann et al., 2009). Future studies need to be conducted to fully understand how a complete neural network in the brain code self-motion during spatial navigation.

## Conclusion

Convergent evidence from behavioral, neurophysiological and computational studies reveals that the vestibular system plays a critical role in different aspects of self-motion perception, such as heading, path, and traveled distance or time. Particularly for heading estimation, a series of physiological studies have been conducted in recent years to address the underlying neural mechanisms. These studies have provided us with valuable information about how the brain may code motion signals to guide spatial navigation. At the same time, these studies also provoke many important issues to be addressed in the future.

First, vestibular signals are widely distributed in the central nervous system. Recent studies have revealed many areas conveying robust vestibular signals in the cerebral cortex. It is likely that more areas will be continually discovered in the future. Thus, it is important to address both the homogeneity and heterogeneity of the functional implications of each area in self-motion perception.

Second, the temporal dynamics of vestibular signals, especially those arise from the otolith organs, vary broadly in the central nervous system. Future studies need to identify exact functions of the neurons with different temporal dynamics. For example, it has been proposed that the momentary vestibular acceleration evidence could be accumulated by decision making neurons e.g., LIP neuron to generate the final behavioral output for heading discrimination task (Drugowitsch et al., 2014). In contrast, velocity information may be used for other functions such as distance perception, or maintenance of visual stability during head or body movements.

Third, vestibular signals arising from the inner ears are encoded in a head-centered reference frame, yet spatial navigation in the environment is basically a body-centered behavior. Recent neurophysiological studies have provided evidence suggesting that vestibular reference frame may be gradually transformed along the signal propagation pathway, for example, from largely head-centered in the rostral regions of the VN (Shaikh et al., 2004), to mixed head- and body-centered in the cerebellar rFN (Kleine et al., 2004; Martin et al., 2018) and the cerebral PIVC (Chen et al., 2013a), and to predominantly body-centered in the cortical area of VIP (Chen et al., 2013a). Future studies need to explore the possible role of neurons with gain modulated activity in reference frame transformation (Zipser and Andersen, 1988; Siegel, 1998; Xing and Andersen, 2000; Gu et al., 2006; Pesaran et al., 2006; Fetsch et al., 2007; Chen et al., 2013b; Hadjidimitrakis et al., 2014; Fan et al., 2015; Yang and Gu, 2017).

Fourth, vestibular signals have been recently discovered in a number of sensory cortices that also carry robust visual motion signals, suggesting that interactions between sensory modalities may exist. It is possible that vestibular and visual signals are integrated by the brain for more robust heading estimate. In addition, it is also possible that these signals may interact with each other for other functions such as maintaining visual stability when smooth pursuit eye movements are accompanied during head or body movements. Future works need to explore these potential functions, as well as the computational rules underlying the integration or interaction process.

Finally, the current review article focuses data mainly collected in the passive self-motion conditions. However, researchers have shown that active self-motion largely diminishes vestibular activity in the brainstem and cerebellum (see review Cullen and Taube, 2017). Recent theoretical studies suggest that a single sensory internal model can combine motor commands with the vestibular and proprioceptive signals optimally to recover accurate self-motion during active head movements (Laurens and Angelaki, 2017). Thus, it would be important to explore the vestibular signals in the cerebral cortex, including sensory cortices, sensory-motor transformation areas, and motor areas under active self-motion conditions.

## Author Contributions

ZC and YG wrote, revised and finalized the manuscript.

## Conflict of Interest Statement

The authors declare that the research was conducted in the absence of any commercial or financial relationships that could be construed as a potential conflict of interest.
